# The meningitis outbreak returns to Niger: Concern, efforts, challenges, and recommendations

**DOI:** 10.1002/iid3.953

**Published:** 2023-07-27

**Authors:** Olivier Sibomana, Clyde Moono Hakayuwa

**Affiliations:** ^1^ Department of General Medicine and Surgery, School of Medicine and Pharmacy College of Medicine and Health Sciences, University of Rwanda Kigali Rwanda; ^2^ Department of Public Health Michael Chilufya Sata School of Medicine Kitwe Zambia

**Keywords:** African meningitis belt, meningitis, Niger, outbreak, vaccination

## Abstract

Meningitis, a disease that commonly manifests in African meningitis belt, continues to be a public health problem as it is a fatal disease that leave survivors with long‐term effects. Most cases of meningitis are due to bacterial and viral infection, although parasites, fungus, cancer, drugs, and immune disorders can rarely cause meningitis. Stiff neck, high temperature, light sensitivity, disorientation, headaches, and vomiting are the most typical symptoms of meningitis. Niger, being in African meningitis belt, has been affected by many meningitis outbreaks. Since 2015, a total of 20,789 cases and 1369 fatalities (CFR 6.6%) have been documented in Niger. In contrast to earlier seasons, the current outbreak of meningitis in Niger exhibits both an increase in the number of cases and a rise in the growth rate. A total of 559 cases of meningitis, including 18 fatalities (overall CFR 3.2%), were reported in the Zinder Region, southeast of Niger, from 1 November 2022 to 27 January 2023, compared to 231 cases reported from 1 November 2021 to 31 January 2022. In the current outbreak, the *Neisseria meningitidis* serogroup C (NmC) is responsible for the majority of laboratory confirmed cases (104/111; 93.7%). To organize the response to the outbreak, a global team of WHO and other partners, including MSF and UNICEF, has been sent to Niger. Even though there are many challenges in battle against meningitis in Niger, immunization, antibiotics administration and strong disease surveillance are recommended techniques to cope with the current meningitis outbreak in Niger.

## Graphical abstract

Meningitis, a disease that commonly manifests in African meningitis belt, continues to be a public health problem as it is a fatal disease that leave survivors with long‐term effects. A total of 559 instances of meningitis, including 18 fatalities (overall CFR 3.2%), were reported in the Zinder Region, southeast of Niger, from November 1, 2022 to January 27, 2023, compared to 231 cases reported from November 1, 2021 to January 31, 2022. Even though there are many challenges in battle against meningitis in Niger, immunization, antibiotics administration and strong disease surveillance are recommended techniques to cope with the current meningitis outbreak in Niger.

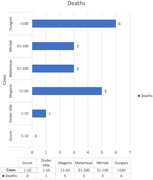

## 1 INTRODUCTION

Meningitis, a fatal disease that leaves survivors with serious long‐term effects, continues to be a serious global public health problem.^1^, ^2^, ^3^ There are threats from cases and outbreaks in many nations around the world. The condition, which is inflammation of the membranes surrounding the brain and spinal cord, is primarily caused by bacterial and viral infection. Meningitis can also be the result of parasitic and fungal infections, and cryptococcal meningitis is more prevalent in HIV positive individuals. Noninfectious causes such as specific drugs, cancer, and autoimmune disorders can also cause meningitis.^1^

Bacterial meningitis has significant long‐term repercussions and a high case fatality rate, and is caused by several kinds of bacteria. These bacteria include Streptococcus pneumoniae, Haemophilus influenzae, *Neisseria meningitidis* (NmA), and *Streptococcus agalactiae* (group B streptococcus). Although it occurs less commonly, other bacteria such as nontyphoidal salmonella, Listeria monocytogenes, Streptococcus suis, and pathogens such as *Staphylococcus aureus* or *Staphylococcus epidermidis* can also cause meningitis.^1^, ^4^

In the African meningitis belt, which Lapeyssonnie first described in 1963, meningococcal meningitis is a major threat.^5^ Meningitis seasonal hyperendemicity and recurrent large‐scale outbreaks are known features of the African meningitis belt, which spans sub‐Saharan Africa from Senegal to Ethiopia.^6^ The epidemic begins early in the dry season and ends quickly with the arrival of the rains, but may recur in the next dry season. Meningitis epidemics mostly last between 2 and 3 years in any given nation. In addition to being difficult to forecast, the recurrence of these epidemics is poorly understood. Meningococcal disease outbreaks are currently controlled by early diagnosis of the disease using the epidemic threshold of ten to fifteen cases per 100,000 people per week, followed by widespread administration of polysaccharide vaccines.^7^

Meningitis epidemics were primarily caused by serogroup A NmA until the development and administration of meningococcal serogroup A conjugate vaccine (MenAfriVac) in the meningitis belt starting in 2010, however since then, no NmA epidemics have happened. Serogroups W (NmW), C (NmC) and X (NmX) have, however, frequently caused epidemics since 2000, sometimes with local incidence rates which can be compared to NmA outbreaks. The causes of epidemics are still speculative, but their identification would improve epidemic prediction and aid in the development of control measures such as immunization.^8^

The incubation period of meningococcal meningitis mostly lasts 4 days on average, however, it can last anywhere between 2 and 10 days.^4^ Stiff neck, high temperature, light sensitivity, disorientation, headaches, and vomiting are the most typical symptoms of meningitis.^9^ Even with prompt diagnosis and appropriate care, 5% to 10% of individuals pass away, usually 24−48 h after their symptoms first appear. 10%−20% of survivors of bacterial meningitis may experience brain damage, hearing loss, or learning disability. Meningococcal septicaemia, which is characterized by a hemorrhagic rash and rapid circulatory collapse, is a less common but much more severe and often fatal variety of the disease.^10^

Person‐to‐person transmission of *N. meningitidis* requires direct contact or respiratory droplets. Since respiratory droplets are susceptible to desiccation and frequently protected by a polysaccharide capsule, close contact is required for human‐to‐human transmission. For example, when two people have personal contact, such as while sharing a drink or kissing, transmission typically occurs through saliva. A human case or carrier, incubator touch, convalescent carrier, or throat carrier could all be the source of infection.^11^

Investigations, a clinical exam, and the patient's medical history are all used to make the diagnosis of meningitis. The Brudzinski sign and the Kernig sign are clinical signs that are strongly indicative of meningitis in the patient. If meningitis is thought to be present, lumbar puncture and cerebrospinal fluid examination are required. Increased opening pressure (>180 mm water), WBCs between 10 and 10,000 cells/L (mostly neutrophils), and low glucose concentration (45 mg/dL) are CSF analytical indicators of meningitis. Furthermore, other gold standard techniques for the diagnosis of meningitis include PCR and culture.^11^

## 2 EPIDEMIOLOGY AND OUTBREAK OF MENINGITIS IN NIGER

**Figure 1:**
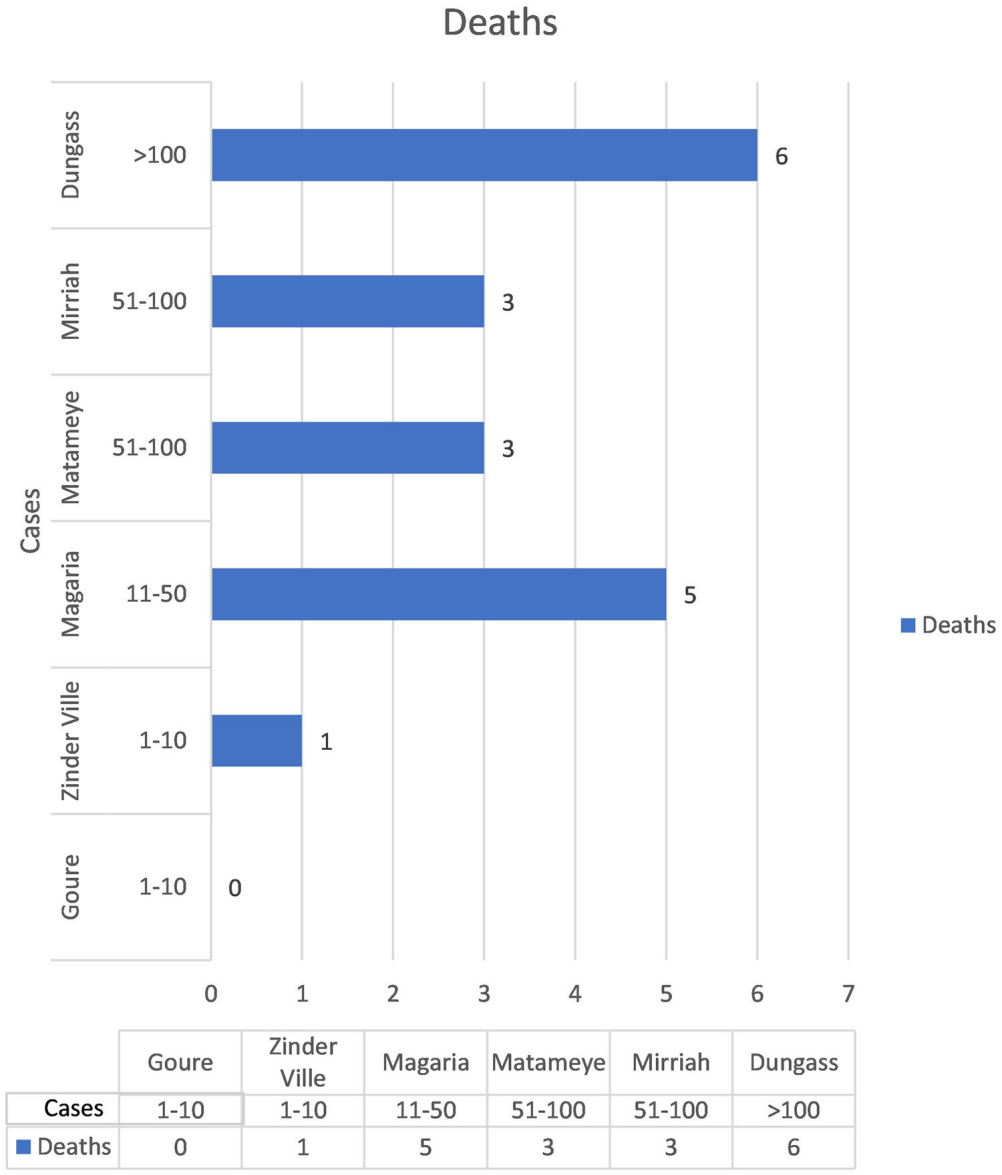
Distribution of reported meningitis cases and deaths by health district, Zinder region, Niger, November 1, 2022−January 27, 2023. *Source*: WHO Health Emergencies Program, World Health Organization.

Niger has been impacted by many meningitis outbreaks due to its location in the African meningitis belt, resulting in 20,789 cases and 1369 fatalities (CFR 6.6%) documented since 2015. A total of 559 instances (111 laboratory confirmed cases) of meningitis, including 18 fatalities (overall CFR 3.2%), have been reported in the Zinder region, southeast of Niger, from November 1, 2022 to January 27, 2023, compared to 231 cases reported from November 2021 1, to January 31, 2022. NmA serogroup C (NmC) is responsible for the majority of laboratory‐confirmed cases (104/111; 93.7%).^10^ Figure 1 shows cases of meningitis reported in Niger by month from October 1, 2021 to January 27, 2023.

Meningitis epidemics occur seasonally in Niger every year due to its location mostly within the African meningitis belt. These outbreaks have been associated with climatic and nonclimatic factors. In dry seasons, outbreaks are common. The dry winds of the Harmattan blow throughout Western Africa around this time, carrying dust and sand particles that irritate the upper respiratory tract mucous membranes of the residents. In fact, the human oropharynx serves as an ecological niche for NmA. A carriage state is when bacteria are colonizing the upper respiratory tract without causing illness. On the other hand, if the oropharyngeal mucosa is injured, such as by a sandstorm, the germs may enter the bloodstream and lead to illness.^12^ The season of meningitis has been observed to end at the beginning of the rainy season, which can also be explained by a decrease in invasiveness presumably caused by less uncomfortable circumstances for the pharyngeal mucosa.^13^ According to a recent study, mean temperature variability has nearly tripled in the last 30 years, and the risk of meningitis has increased globally by 4.8%.^14^ Nonclimatic risk factors for meningitis outbreaks include poor housing condition and household overcrowding, low economic index of well‐being, age (high risk for young children), respiratory tract and viral infections, smoking, poor health care seeking behavior, inadequate vaccination and vaccine hesitancy.^15^, ^16^, ^17^ Furthermore, population mixing, the simultaneous presence of other epidemics such as measles, diphtheria, and COVID‐19 in the same region, insecurity, and displacement of the population, all of which occur in the context of a prolonged humanitarian crisis, are likely to contribute to the spread of the outbreak.^10^

In contrast to earlier seasons, the current outbreak exhibits both an increase in the number of cases and an increase in the growth rate. In contrast to the most recent meningitis outbreak in the Zinder region which had a total of 372 cases during the 2021–2022 season, a total of 559 cases of meningitis were recorded in only three months, from November 1, 2022 to January 27, 2023. The risk of an international spread is confirmed by the fact that Jigawa State in Nigeria, where a NmC outbreak is also ongoing, and Zinder Region share a border internationally. Furthermore, the concurrent incidence of other epidemics, insecurity, and population relocation, all within the framework of a prolonged humanitarian crisis, are likely to aid in the spread of the outbreak to other subregional nations in West Africa.^10^

## 3 EFFORTS TO CONQUER MENINGITIS IN NIGER

In the Zinder area, a technical committee has been formed to organize the response to the pandemic. To help in response, a global team from WHO and other partners, including MSF and UNICEF, has been sent out. Case investigations are part of the surveillance system operations that have been strengthened in the Zinder region, particularly in the Dungass health district. Laboratories are still collecting samples and confirming results of probable meningitis cases. Acquisition of antibiotic ceftriaxone, isolation of patients, deployment of health workers for case management, dissemination of case management guidelines, and free treatment for cases are only a few of the case management actions that have been strengthened.^10^

The International Coordinating Group on Vaccine Provision approved and delivered a request for 608 960 doses of the trivalent ACW polysaccharide vaccine on December 31, 2022, and January 9, 2023, in two batches of roughly 300,000 doses each. The Global Alliance for Vaccines and Immunization and WHO have supported the Ministry of Health in implementing reactive vaccination campaigns with the trivalent ACW meningococcal polysaccharide vaccine in the health districts of Dungass, Gouré, Mirriah, and Matamèye, targeting the age range of 2−29 years. Overall, a 99.8% immunization rate was achieved. In close collaboration with administrators and community leaders in affected districts, risk communication and community engagement activities are ongoing, providing health advice and infection, prevention, and control recommendations through community radios and other channels, including door‐to‐door education on the necessity of seeking immediate medical attention if symptoms occur to promptly begin treatment.^10^

## 4 CHALLENGES TO FIGHT MENINGITIS IN NIGER AS WELL AS IN AFRICAN MENINGITIS BELT

Challenges in conquering meningitis in African meningitis belt is found in prevention, epidemic control, diagnosis, treatment, and disease surveillance. First, multivalent conjugate vaccines are inconsistently used, rarely available, expensive, and some serogroups are not covered by existing vaccines.^1^ Furthermore, the lack of resources, such as laboratories, equipment, and qualified employees, as well as the availability of drugs and money, are significant impediments in low‐and middle‐income countries (LMICs), especially in the African meningitis belt.^18^

Meningococcal diseases manifests as sporadic cases or outbreaks and is dynamic and highly unpredictable.^19^ Although a higher percentage of laboratory confirmation in cases during and between epidemics can help assess the spread and threat of new clones, vaccines are difficult to obtain due to the unpredictable nature of epidemics and the pathogens involved, as well as the long cycle of vaccine production and the short shelf life of vaccines.^1^

In many nations, ceftriaxone, a highly efficient antibiotic, is the standard course of therapy for meningitis.^20^ The availability of ceftriaxone in the African meningitis belt is limited, which may result in less‐than‐ideal treatment plans. Lack of access to care causes delays in treatment initiation, which in turn causes subpar results. Empiric treatments are frequently used due to limited microbiological capabilities and a lack of accessible and affordable diagnostics for diagnosis. Furthermore, in LMICs, health care community workers may not be aware of the value of screening for acute problems, such as seizures and symptoms of elevated intracranial pressure, as well as sequelae, particularly if there are no approved treatments.^1^

Meningococcal disease must be controlled through surveillance, with outbreak detection, incidence monitoring, disease burden estimation, analyses of antibiotic resistance, evaluations of control strategy, and serogroup and strain distribution assessments serving as the main drivers of surveillance networks.^21^, ^22^ Surprisingly, there are still large gap in meningitis disease surveillance in terms of policies and financial support. Several regions do not prioritize meningitis disease surveillance leading to lack of national guidance for its implementation, and most low‐income countries still rely on external financial support to conduct surveillance.^1^

With seven and six children per woman, Niger is one of the nations with the highest fertility rates worldwide. With 5 million residents, or 1/5 of the entire population, Zinder is the region with the highest population in Niger. The effects of this high population in the Zinder region include unemployment, poverty, drug use and violence.^23^ In Zinder, entire villages are constantly experiencing crises, mostly as a result of drought, price shocks, extreme population pressure and climate change. The lack of access to land, agricultural inputs and water, as well as lack of income sources and assets, contribute to the inaccessibility of food, causing a dramatic increase of malnutrition.^24^ Periodic famines, terrible poverty, and a high rate of crime, especially gender‐based violence, undereducated population, and the trafficking of narcotics, guns, and people, are all problems in Zinder.^25^ These problems, particularly overpopulation, poverty, undereducation and humanitarian crises, are specific challenges to combat meningitis in the Zinder region of Niger.

## 5 RECOMMENDATIONS

The most efficient strategy to reduce the severity and effects of meningitis is to prevent it through immunization, which provides long‐lasting protection.^26^ A public health priority is the distribution of multivalent conjugate meningococcal vaccines to prevent bacterial meningitis epidemics throughout the African meningitis belt. To prevent the return of epidemics, routine immunization programs and maintaining high vaccination coverage are essential.

When promptly administered, antibiotics play an important role in the treatment of meningitis. The right antibiotics must be administered as soon as possible. Meningitis is treated with a variety of medications, such as penicillin, ampicillin, and ceftriaxone. Ceftriaxone is the recommended medication for meningococcal and pneumococcal meningitis epidemics. It is best to perform a lumbar puncture first since antibiotics may make it more difficult for germs to grow in spinal fluid. Blood sampling, however, can also be useful in determining the causative agent, and the priority is to begin treatment early.^10^, ^27^

Antibiotic prophylaxis (AP) can be used to efficiently prevent infection, but to avoid excessive expense, toxicity, and antibiotic resistance, its use should be restricted to certain well‐accepted purposes. Antimicrobial prophylaxis can be used to prevent infection by eradicating a colonizing organism. It can also be used to prevent infection by preventing the recurrence or reactivation of an existing infection.^28^ Chemoprophylaxis for meningitis is advised for close household connections outside the meningitis belt in Africa. For close contacts in the meningitis belt, chemoprophylaxis is recommended in nonepidemic circumstances.^10^, ^29^

Appropriate case management, proactive community case finding, and reactive mass immunization of affected populations constitute the response to the epidemic. Meningitis must be controlled through surveillance, from case identification to inquiry and laboratory confirmation.^10^ Health care professionals and laboratories should be informed of any prospective meningococcal outbreaks, urged to look for any symptoms, and instructed to report any cases as soon as possible. The CDC recommended that case referral and additional confirmatory tests be performed if the disease is suspected while the lab results are negative.^11^

## 6 CONCLUSION

Due to its high fatality rate and the potential for severe long‐term consequences, meningococcal meningitis continues to be a public health concern, especially in the African meningitis belt. The current meningitis outbreak in Niger is of a great concern. Rapid measures including mass immunization, screening, drug administration and disease surveillance are recommended to be implemented by Niger government and other countries which are particularly in African meningitis belt. Nations, international organizations, vaccine industries, epidemiology experts and NGOs are called to work together to eradicate meningitis in Niger and other countries in African meningitis belt.

## AUTHOR CONTRIBUTIONS

**Olivier Sibomana**: Conceptualization, methodology, project administration, supervision, validation, writing—original draft. **Clyde Moono Hakayuwa**: Software, writing‐review and editing. All authors: Approving the final draft.

## CONFLICT OF INTEREST STATEMENT

The authors declare no conflict of interest.
